# A Compound Comminuted Fracture of the Frontal Bone, with Loss of Brain Substance—Recovery

**Published:** 1887-04

**Authors:** R. C. Nettles

**Affiliations:** Marlin, Texas


### A COMPOUND COMMINUTED FRACTURE OF THE FRONTAL BONE, WITH LOSS OF BRAIN SUBSTANCE—RECOVERY.

*By R. C. Nettles, M. D., Marlin, Texas.*

For Daniel's Texas Medical Journal.

BENNIE PIERSON, a deaf mute, aged 6 years 3 months, was with his father in the lot about 7 o'clock on the morning of the 8th of November, 1886, Going up behind and striking a mule colt he was kicked in the forehead, just above the nasal eminence, suffering a compound comminuted fracture of the frontal bone. Upon going to him his father found him getting up, somewhat stunned, but conscious. He was borne into the house and laid upon a pallet. Dr. J. C. Daniel, the family physician, saw him in less than an hour. He found a gaping wound, which had bled freely and was filled with clotted blood, cntangling some brain substance. Recognizing the gravity of the accident, without further examination he asked for surgical counsel and assistance.

He says the little fellow was entirely conscious, but owing to his deafness he could not converse with him. He was restless and had vomited four or five times. He noticed slight twitchings of some of the muscles, but no convulsions. He administered ⅙ grain morphine, which quieted him.

I reached the patient at 2 p. m. and found Dr. Whatley also with Dr. Daniel. The patient was lying quietly asleep. When touched he opened his eyes and looked around intelligently. We first washed his face and forehead, against which he protested vigorously, getting up on his feet and requiring to be held in his fathers arms. Dr. Whatley administered chloroform. Upon examining the wound we found it situated about three quarters of an inch above the eyebrows, in length two inches, crescentic in shape, like the toe of the colt's hind foot. Entangled in the clotted blood filling the wound, there was about a fluid drachm of gray brain substance.

Clearing out the wound, we found the frontal bone fractured, and the lower edge of the fractured portion driven backward a half inch. Failing to get this in position with the elevator, without endangering the membranes on the rough edge of the fractured bone, Dr. Daniel assisting me, I enlarged the scalp wound, and applying the trephine on the sound bone, took out a three quarter button, which permitted the depressed portion to be easily elevated. Finding the piece completely fractured all around, and simply held in position by an attachment of dura mater above, not sufficient to maintain its nutrition, we removed it entirely, with several fragments and spiculæ found under it. In the left angle of the wound the membranes showed a small laceration, with clots in the mouths of the wounded arteries. A sharp crust of bone was made smooth with the forceps, a drainage tube laid across the wound, with its ends emerging at the angles, and the scalp was adjusted with silk interrupted sutures. The whole operation was done in the strictest antiseptic mode, we using solutions of carbolic acid 1 to 20, and corrosive sublimate 1 to 2000. The wound was dusted with iodoform, over which was applied, 1st iodoform gauze, 2nd corrosive sublimate gauze, with antiseptic cotton and rubber protective, and this dressing secured with a roller bandage.

The piece of bone removed was two inches long, by one inch wide, and had across its center the markings of the frontal suture.

One hour after the operation the patient's pulse was 106. On the 9th he had fever, pulse 135, temperature not taken. Dr. Daniel administered drop doses of tinct. aconite until eight doses had been given, when fever subsided and pulse came down to 110, above which it did not afterwards go. On the morning of 10th, pulse was 100. That night he had slight fever and was restless. He was given 1/6 grain morphine. After this he had no more fever, nor medicine of any kind. His bowels were moved by enema once. On the 10th he was up and could not be kept in bed. On account of being deaf he could not be made to understand the situation and the necessity of being quiet.

I visited the patient again on the seventh day. He was running about the house and met me at the door. The first dressing had not been disturbed. Upon removing it there was a small amount of broken down brain substance in the drainage tube in the left angle of the wound. There was no pus, nor bad odor. The absorbent and antiseptic dressing was stained reddish with the sanguinolent oozing, and the scalp wound was entirely healed.

The dressing was applied again antiseptically as before, the drainage tube being left in position. This second dressing was removed one week afterwards by Dr. Daniel, who subsequently had the patient under observation. The drainage tube was kept in the wound three weeks, when it was withdrawn, and the wound healed up kindly, leaving a space like a large fontanelle. He has suffered no symptoms up to the present time and is now quite well.

*Marlin, Texas, March 31, 1887.*

